# Activation of retinoic acid-related orphan receptor γ(t) by parabens and benzophenone UV-filters

**DOI:** 10.1016/j.tox.2022.153159

**Published:** 2022-03-23

**Authors:** Silvia G. Inderbinen, Manuel Kley, Michael Zogg, Manuel Sellner, André Fischer, Jacek Kędzierski, Stéphanie Boudon, Anton M. Jetten, Martin Smieško, Alex Odermatt

**Affiliations:** a Division of Molecular and Systems Toxicology, Department of Pharmaceutical Sciences, University of Basel, Klingelbergstrasse 50, 4056 Basel, Switzerland; b Swiss Centre for Applied Human Toxicology and Department of Pharmaceutical Sciences, University of Basel, Missionsstrasse 64, 4055 Basel, Switzerland; c Computational Pharmacy, Department of Pharmaceutical Sciences, University of Basel, Klingelbergstrasse 61, 4056 Basel, Switzerland; d Immunity, Inflammation, and Disease Laboratory, National Institute of Environmental Health Sciences, National Institutes of Health, 111. T.W. Alexander Drive, Research Triangle Park, NC 27709, USA

**Keywords:** Retinoic acid-related orphan receptor gamma, Paraben, UV-filter, Th17 cell, Immune disease, Inflammation

## Abstract

Retinoic acid-related orphan receptor γt (RORγt) regulates immune responses and its impaired function contributes to inflammatory and autoimmune diseases and may promote skin cancer. Synthetic inverse RORγt agonists block the production of Th17-associated cytokines including interleukin (IL)-17A and IL-22 and are under investigation for treatment of such pathologies. Unintentional RORγt activation in skin, following exposure to environmental chemicals, may promote inflammatory skin disease. Parabens and UV-filters, frequently used as additives in cosmetics and body care products, are intensively inspected for endocrine disrupting properties. This study assessed whether such compounds can interfere with RORγ activity using a previously established tetracycline-inducible reporter gene assay in CHO cells. These transactivation experiments revealed hexylparaben, benzylparaben and benzophenone-10 as RORγ agonists (EC_50_ values: 144 ± 97 nM, 3.39 ± 1.74 μM and 1.67 ± 1.04 μM, respectively), and they could restore RORγ activity after suppression by an inverse agonist. Furthermore, they enhanced RORγt-dependent transcription of the pro-inflammatory IL-17A and/or IL-22 genes in the murine T-cell model EL4. Virtual screening of a cosmetics database for structurally similar chemicals and *in vitro* testing of the most promising hits revealed benzylbenzoate, benzylsalicylate and 4-methylphenylbenzoate as RORγ agonists (low micromolar EC_50_ values). Moreover, an analysis of mixtures of the newly identified RORγ agonists suggested additive effects. This study presents novel RORγ(t) agonistic structural scaffolds. By activating RORγ(t) the identified parabens and UV-filters may potentially aggravate pathophysiological conditions, especially skin diseases where highest exposure of such chemicals can be expected. Follow-up studies should assess whether such compounds, either alone or as mixtures, can reach relevant concentrations in tissues and target cells to activate RORγ(t) *in vivo*.

## 1. Introduction

Retinoic acid-related orphan receptors (RORs) are nuclear receptors acting as ligand-dependent transcription factors. RORs are involved in multiple physiological functions, including lipid and glucose metabolism, immune response, and circadian rhythm (Jetten, 2009). RORγ and its shorter isoform RORγt share the same ligand binding domain (LBD) and are expressed in distinct cells and tissues, thereby exerting different biological functions (He et al., 1998; Villey et al., 1999). RORγ is widely expressed in peripheral tissues, including liver, adipose tissue, skeletal muscle and kidney (Hirose et al., 1994; Jetten et al., 2013; Medvedev et al., 1996). In contrast, RORγt is specifically expressed in a group of immune cells, including T helper 17 cells (Th17) and innate lymphoid cells 3 (ILC3) (Ivanov et al., 2006; Montaldo et al., 2015), and was found to be associated with enhanced inflammation in autoimmune disorders, such as psoriasis, rheumatoid arthritis, multiple sclerosis and Crohn’s disease (Miossec and Kolls, 2012). For example, in mouse psoriasis models RORγt positive ILC3 account for the production of the proinflammatory IL-17A and the potent keratinocyte growth factor IL-22 (Pantelyushin et al., 2012) and levels of this cell type are increased in human psoriatic skin (Cai et al., 2011; Villanova et al., 2014). Upregulation of RORγt and elevated levels of IL-17A and IL-22 producing tissue-resident memory CD8 positive T cells have been observed in skin from patients with psoriatic arthritis (Leijten et al., 2021). Furthermore, chronic UV-radiation exposure induces a local immune shift toward RORγt positive IL-17A/IL-22 producing ILC3 that are involved in mutant skin cell growth, thus promoting skin cancer (Lewis et al., 2021).

Mice lacking RORγt were protected against the development of Th17-dependent inflammation in autoimmune disease models (Ivanov et al., 2006; Yang et al., 2008). This raised the interest for RORγt as potential drug target to treat such disorders and several small molecules inhibiting RORγt have been developed and were shown to ameliorate autoimmune and inflammatory pathologies in animal models (reviewed in Fauber and Magnuson, 2014; Jetten and Cook, 2020; Sun et al., 2019). For instance, treatment with RORγt inhibitors reduced the expression of IL-17A and IL-22, which stimulated the release of proinflammatory cytokines and chemokines from primary human lung cells and in allergic airway inflammation in mice (Chung et al., 2006; Lamb et al., 2021; Pan et al., 2013; Zheng et al., 2007). Additionally, natural compounds such as ursolic acid and digoxin and some azole antifungals were found to inhibit RORγt, thereby reducing IL-17A gene expression (Huh et al., 2011; Kojima et al., 2012; Xu et al., 2011).

RORs are thought to be constitutively active, suggesting that coactivators can activate ROR-mediated gene expression even in the absence of active ligands (Solt et al., 2010), or that they are constantly activated by endogenous (not yet identified) ligands present in cultured cells. Nevertheless, basal RORγ(t) activity can be further enhanced by agonists interacting with the LBD (Strutzenberg et al., 2019). In contrast to numerous inhibitors, only few studies described exogenous substances that activate RORγ(t), including the synthetic SR1078 (Wang et al., 2010) and SR0987 (Chang et al., 2016), and natural compounds such as digoxigenin (Karaś et al., 2018) and isoflavones (Kojima et al., 2015).

The effects of RORγt activators have not been fully elucidated and are likely highly tissue- and context-dependent. Their benefit in the support of the host defense against bacterial and fungal infections that are defeated by Th17 cell-dependent mechanisms has been suggested (Kojima et al., 2015; Solt and Burris, 2012). Other reports postulated their potential in cancer treatment, with the strategy of using Th17 cells in adaptive immune cancer therapy (Bailey et al., 2014; Chang et al., 2016; Hu et al., 2016; Martin-Orozco et al., 2009). However, by enhancing Th17 cell-dependent pro-inflammatory mediators, RORγt agonists may aggravate inflammatory and autoimmune diseases. Thus, it is important to identify xenobiotics that may lead to unintentional RORγt activation.

Body care products and cosmetics contribute considerably to the exposure of humans to exogenous chemicals. Many of these products contain several paraben and UV-filter chemicals and their potential endocrine disrupting effects are of broad concern, widely studied but controversially discussed (Darbre and Harvey, 2014; Krause et al., 2012; Matwiejczuk et al., 2020; Nowak et al., 2018; Wang et al., 2016). In contrast, less is known on potential immune disrupting effects and xenobiotics-induced RORγ activation may disrupt immune responses. The U.S. Tox21 program included a quantitative high-throughput screening to detect small molecule antagonists of RORγ (PubChem, 2015), suggesting that some parabens and benzophenone UV-filters might modulate the activity of this receptor. However, the data was inconclusive due to technical limitations of the high-throughput screening approach and some of the tested parabens and benzophenone UV-filters were cautiously suggested as potential RORγ agonists, without further elucidation.

Parabens have a broad spectrum of preservative effects and they are widely used as additives in cosmetics, medicines and processed foods to maintain long-term stability (CIR, 2008; Nowak et al., 2018). Parabens are esters of *p*-hydroxybenzoic acid (also described as 4-hydroxybenzoic acid) characterized by low production costs, excellent chemical stability and generally considered to be well tolerated (Matwiejczuk et al., 2020). Nevertheless, based on *in vitro* studies, parabens have been associated with disturbances of estrogen receptor function and estrogen metabolizing enzymes (Boberg et al., 2010; Darbre and Harvey, 2008; Engeli et al., 2017). Additionally, anti-androgenic properties of parabens were reported and a recent study described interferences with additional nuclear receptors (Chen et al., 2007; Ding et al., 2017; Fujino et al., 2019; Satoh et al., 2005). The relevance of these observations regarding human exposure scenarios remains to be investigated. Exposure to parabens mainly takes place through oral intake and transdermal absorption. Due to the rapid hepatic and intestinal hydrolysis, topical application of paraben containing cosmetic products is considered as the main route of human exposure, with a higher contribution to circulating levels compared to oral intake (Matwiejczuk et al., 2020).

Most sunscreens contain high levels of several UV-filter chemicals that can absorb and dissipate UV-radiation to protect the skin of the users from sunburn, ultimately preventing skin aging and skin cancer (Bens, 2014). Additionally, UV-filters are additives in multiple cosmetic products such as hair spray, shampoo, make-up, perfumes, and skin care products to protect ingredients from the effects of UV-radiation and consequently enhance product stability and durability (Liao and Kannan, 2014). An increasing number of human cell- and yeast-based *in vitro* studies and animal investigations suggest that some organic UV-filters can cause endocrine disrupting effects, including estrogenic and androgenic disturbances, as well as disturbances of thyroid hormone- and progesterone receptor-mediated signaling (reviewed in Krause et al., 2012; Kunz and Fent, 2006; Schlecht et al., 2004; Wang et al., 2016). Similar to parabens, the main route of human exposure to UV-filters is *via* dermal uptake after topical application of sunscreens and cosmetics, allowing direct entrance to the systemic circulation without first-pass effect in the liver (Krause et al., 2012; Sarveiya et al., 2004). Importantly, human exposure to UV-filters is not limited to summer and usage of sun cream, suggesting that a substantial exposure derives from other body care products (Calafat et al., 2008).

Considering its important function in skin and preliminary indications from Tox21 data, the present study further tested the hypothesis that RORγ(t) might be modulated by parabens and UV-filters, to which the majority of the human population is regularly exposed, by assessing the effect of such chemicals on RORγ(t) transcriptional activity. To detect additional RORγ(t) modulating chemicals, a computational similarity search was performed using the identified compounds and known RORγ(t) modulators analyzed against a database containing more than 6000 constituents of body care products. Since most cosmetic products contain several parabens and UV-filters, effects of mixtures of the examined compounds on RORγ(t) activity were assessed. Finally, the binding modes of methylparaben and hexylparaben were inspected by computational modeling to deduce structural elements responsible for their diverging activity towards RORγ(t).

## 2. Materials and methods

### 2.1. Chemicals and reagents

GSK2981278, SR2211, SR0987, phorbol 12-myristate 13-acetate (PMA), and ionomycin were purchased from Cayman Chemicals (Ann Arbor, MI, USA), benzophenone-9 and benzophenone-10 from Carbosynth (Compton, UK), and the remaining benzophenone derivatives, 3-benzylidene camphor (3-BC) and 4-methylbenzylidene camphor (4-MBC) from Merck AG (Glattbrugg, Switzerland). All other chemicals were obtained from Sigma-Aldrich (Buchs, Switzerland). RPMI-1640 cell culture medium (R8758) was purchased from Sigma-Aldrich, Ham’s F12 Nutrient Mix (21765–029) from Gibco (Thermo Fischer Scientific, Waltham, MA, USA), fetal bovine serum (FBS, S1810–500) from Biowest (Nuaillé, France) and defined FBS for the use in Tet-on systems (HyClone, SH30070) from Cytiva (Marlborough, MA, USA).

### 2.2. Cell culture

Chinese hamster ovary (CHO) cells stably expressing a doxycycline-inducible RORγ Tet-on system (Slominski et al., 2014) were cultured in Ham’s F12 Nutrient Mix medium supplemented with 10% defined FBS, 100 U/mL penicillin and 0.1 mg/mL streptomycin. RPMI-1640 medium supplemented with 10% FBS, 100 U/mL penicillin, 0.1 mg/mL streptomycin and 50 μM β-mercaptoethanol was used to culture the murine EL4 T-cell line, in suspension in 75 cm^2^ cell culture flasks from Falcon (Corning Inc., Corning, NY, USA). Both cell lines were cultured under standard conditions (37 °C, 5% CO_2_).

### 2.3. RORγ-reporter gene assays

The CHO RORγ Tet-on cell line used in this study is a well-established reporter gene system to assess RORγ agonists and antagonists. This cell model bears a tetracycline-inducible RORγ expression cassette and a ROR response element (RORE)-dependent luciferase reporter gene that is activated upon binding of RORγ to the RORE (Slominski et al., 2014). CHO RORγ Tet-on cells were seeded in 96-well plates at a density of 2000 cells/100 μL culture medium, followed by incubation overnight. Subsequently, the medium was renewed, and the respective test compound dissolved in dimethylsulfoxide (DMSO) was added to the wells using a HP D300 Digital Liquid Dispenser with T8 dispense heads (HP Inc., Palo Alto, CA, USA). In experiments using RORγ inverse agonist, SR2211, was added at a final concentration of 1.25 μM, simultaneously with the test compound. Two hours later, RORγ expression was induced by adding doxycycline at 1 μM final concentration. All samples were adjusted to contain the same volume and concentration of DMSO. After 16 h of incubation, cells were washed with PBS, lysed with 20 μL Tropix lysis solution (Applied Biosystems, Foster City, CA, USA) containing 1 mM dithiothreitol and frozen at − 80 °C for at least 20 min. Luciferase activity was measured with a Cytation 5 reader (BioTek, Winooski, VT, USA) or SpectraMax L Microplate Reader (Molecular Devices, San Jose, CA, USA), injecting 100 μL D-luciferin substrate solution [0.56 mM D-luciferin, 63 mM ATP, 0.27 mM CoA, 0.13 mM EDTA, 33.3 mM dithiothreitol, 8 mM MgSO_4_, 20 mM tricine (pH 7.8)] to 10 μL of corresponding cell lysates, prepared on pure grade white 96-well microtitration plates (BRAND, Wertheim, Germany). Measured values were normalized to values from control cells treated with DMSO vehicle control only and induced with doxycycline.

### 2.4. Measurement of IL-17A and IL-22 mRNA expression in EL4 cells

EL4 cells were transfected with plasmid encoding the human variant of RORγt by electroporation and seeded into 12-well plates (500,000 cells and 1.6 μg plasmid in 2 mL cultivation medium per well). Transfection was performed using the NeonTM transfection system (Invitrogen, Carlsbad, CA, USA) as follows: 1.8 × 10^6^ cells and 6 μg plasmid in 100 μL buffer R were exposed to one pulse (1080 V, 50 ms) utilizing a 100 μL gold tip. After incubation for 16 h, the cells were treated for 5 h with the indicated test compounds as well as PMA and ionomycin at a total concentration of 0.253 nM and 31.3 nM, respectively. Subsequently, cells were centrifuged at 200 *g* for 3 min and total RNA was isolated from the pellet with the RNeasy Mini Kit according to the manufacturer’s protocol (Qiagen, Venlo, Netherlands). Contamination with genomic DNA was prevented by on-column DNA digestion using DNase I (RNase-free, Qiagen). RNA (1 μg) was reverse transcribed to complementary DNA (cDNA) utilizing the Takara PrimeScript RT Reagent Kit, according to the manufacturer’s protocol (Takara Bio Inc., Kusatsu, Japan). Finally, quantitative polymerase chain reaction (qPCR) was performed using Kapa SYBER^®^ Fast qPCR Master Mix (KAPA Biosystems, Wilmington, MA, USA) as described earlier (Inderbinen et al., 2020). The following primers were used: peptidylprolyl isomerase A (PPIA): forward 5’-CAAATGCTGGACCAAACACAAACG-3’, reverse 5’-GTTCATGCCTTCTTTCACCTTCCC-3’; IL-17A: forward 5’-TCAAAGCTCAGCGTGT CCAA-3’, reverse 5’-TCTTCATTGCGGTGGAGAGTC-3’; IL-22: forward 5’-GTGCGATCTCTGATGGCTGT-3’, reverse 5’-TCCTTAGCACTGACTCCTCG-3’. Primer quality was verified by analysis of melting curves and inspection of the PCR product on an agarose gel (single band at the expected size of the amplified sequence). Resulting CT values were normalized to values of the endogenous control gene PPIA and compared to control according to the 2^−ΔΔCT^ method (Schmittgen and Livak, 2008).

### 2.5. Computational similarity search

The computational similarity search was performed using the CosIng cosmetics database published by the European Commission (https://ec.europa.eu/growth/tools-databases/cosing) using several parabens, UV-filters and published RORγ(t) agonists as templates. The following template compounds were selected: hexylparaben (1083–27-8), benzylparaben (94–18-8), butylparaben (94–26-8), phenylparaben (17696–62-7), benzophenone-6 (131–54-4), and SR0987 (303126–97-8). All entries were retrieved from the CosIng database for which a unique CAS number was provided that were neither restricted nor banned. To obtain isomeric SMILES strings for these 10,754 CAS numbers, the Cactus web server of the National Cancer Institute (https://cactus.nci.nih.gov) as well as the PubChem database (Kim et al., 2019) were considered, resulting in 6715 entries. As some CAS numbers represented compound mixtures, their largest organic fragment was retained. A manual inspection was performed for entries that failed to produce unambiguous structural input. This revealed extracts with complex characteristic, entries with missing SMILES strings or incomplete structural information entries.

To compute the Extended Connectivity Fingerprints (ECFP), OpenBabel (v3.0.0) (O’Boyle et al., 2011) was employed and the library was compared to the templates by calculating the Tanimoto coefficient. Further, Extended Three-dimensional Fingerprints (E3FP) were generated using an open-source python package (Swain, 2013). The LigPrep routine within the Maestro Small-Molecule Drug Discovery Suite (Schrödinger LCC, 2019) was selected to assign protonation states with Epik at pH 7.4 and to obtain low-energy conformers using the OPLS3e force field. For each compound, a maximum of five conformers was retained and their highest Tanimoto coefficient was considered as similarity score.

### 2.6. Statistical analysis

Data were analyzed using the RStudio software (RStudio Team 2020: Integrated Development for R. RStudio, PBC, Boston, MA, http://www.rstudio.com/.) using the one-way analysis of variance (ANOVA) followed by the Dunnetts’s post hoc test or Kruskal-Wallis test followed by Dunn’s test to evaluate differences between control and treated cells in reporter-gene assays.

qRT-PCR experiments were analyzed with GraphPad Prism version 5 for Windows (GraphPad Software, San Diego, California USA, www.graphpad.com) performing ANOVA and the Dunnetts’s post-hoc test. For estimation of EC_50_ values, concentration-response curves were fitted and analyzed by non-linear regression and data was additionally analyzed by one-way analysis of variance (ANOVA) followed by Dunnett’s post-hoc test using GraphPad Prism version 5 software as well.

## 3. Results

### 3.1. Identification of parabens and benzophenone UV-filters that activate RORγ

**Fig. 1.**
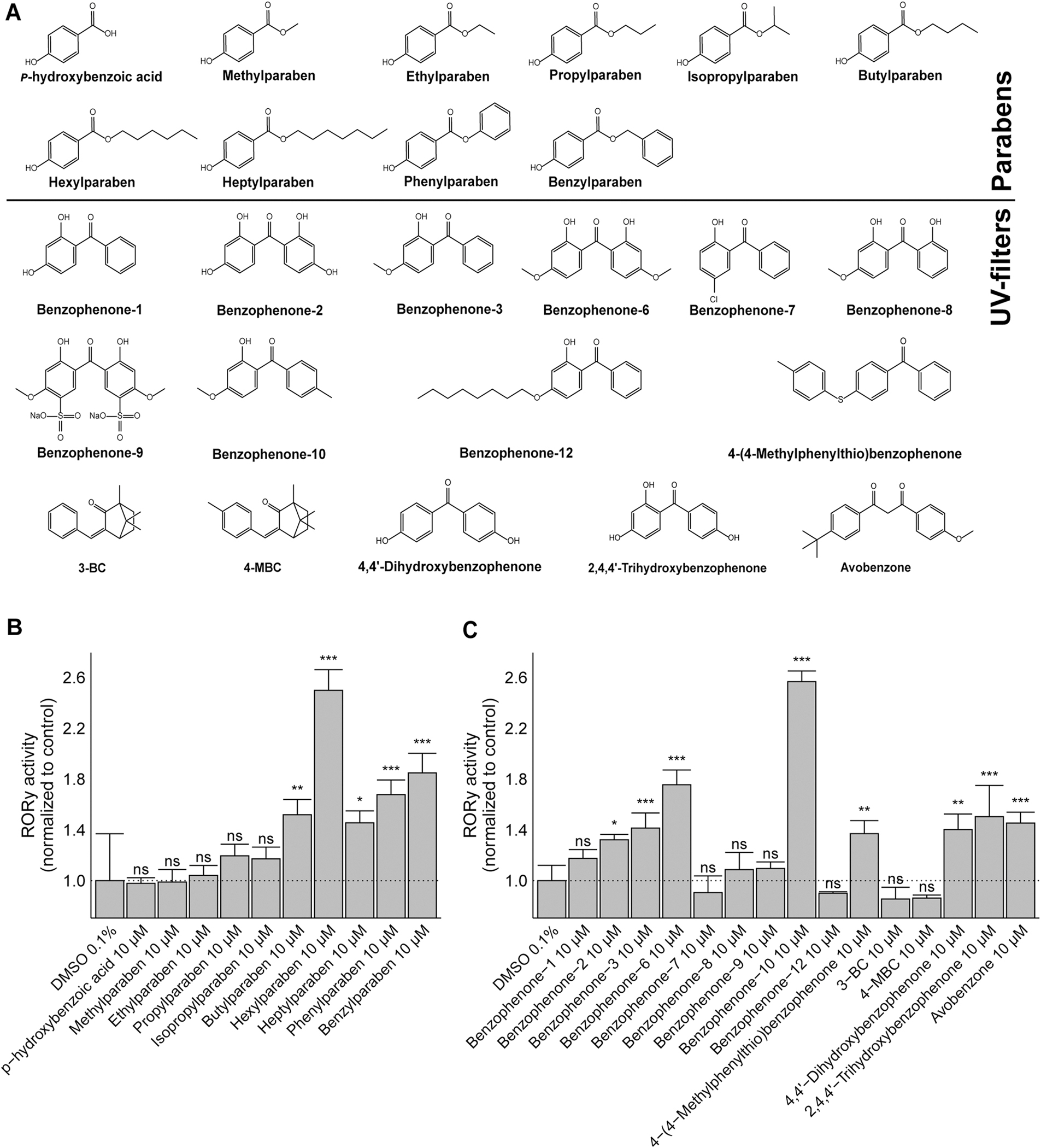
Identification of parabens and UV-filters activating RORγ. **(A)** Chemical structures of parabens and UV-filters tested. **(B)** Activation of RORγ by parabens and **(C)** benzophenone UV-filters. RORγ expression was induced by doxycycline and cells were treated with the test compounds at the concentrations indicated. Luciferase activity was determined and normalized to that of the vehicle control DMSO. Data represent mean ± SD from three independent experiments. Statistical analysis was performed by one-way ANOVA followed by the Dunnett’s post-hoc test. P values: *** < 0.001, ns (not significant).

To assess whether parabens and UV-filters (for structures of examined chemicals see Fig. 1A) can interfere with RORγ activity, an already established doxycycline-inducible CHO RORγ Tet-on cell system was used in a transactivation assay (Slominski et al., 2014). A first screening of parabens at a concentration of 10 μM revealed that benzyl-, hexyl-, phenyl-, heptyl- and butylparaben activated RORγ-dependent transactivation by more than 1.5-fold compared to the DMSO vehicle control (Fig. 1B). The main paraben metabolite *p*-hydroxybenzoic acid and the short-chain length parabens methyl-, ethyl-, propyl- and isopropylparaben were inactive at this rather high concentration. The benzophenone-type UV-filters benzophenone-2, −3, −6 and −10, as well as 4-(4-methylphenylthio)benzophenone, 4,4’-dihydroxy- and 2,4,4’-trihydroxybenzophenone showed agonistic activity at 10 μM.

**Fig. 2.**
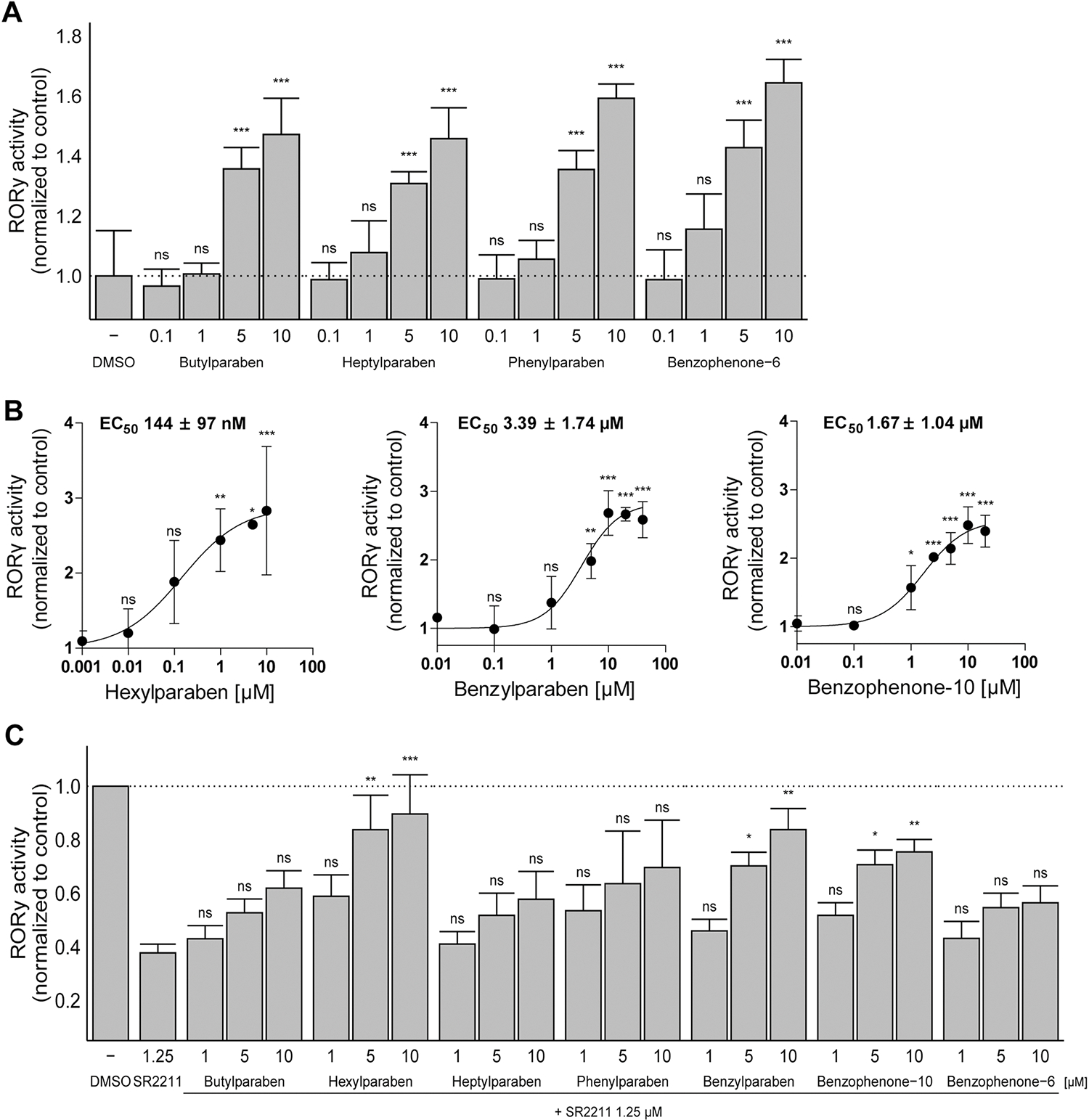
Concentration-dependent agonist and antagonist activities of selected parabens and benzophenone UV-filters towards RORγ. **(A, B)** Concentration-dependent activation of RORγ by parabens and benzophenone UV-filters. RORγ expression was induced by doxycycline and cells were treated with the test compounds at the concentrations indicated. Luciferase activity was determined and normalized to that of the vehicle control DMSO. **(B)** Concentration-response curves were fitted and analyzed by non-linear regression. **(C)** Competition of parabens and benzophenone UV-filters with the RORγ inverse agonist SR2211. RORγ expression was initiated by doxycycline and basal RORγ-dependent transcriptional activity was suppressed by 1.25 μM SR2211. Following incubation with the test compounds at the concentrations indicated, reactivation of the RORγ-dependent reporter gene signal was measured and normalized to that of the DMSO vehicle control. Data represent mean ± SD from at least three independent experiments and were analyzed by (A, B) one-way ANOVA followed by the Dunnett’s post-hoc test or (C) Kruskal-Wallis test with Dunn’s post-hoc test, p values: * < 0.05, ** < 0.01, *** < 0.001, ns (not significant).

Concentration-dependence was then analyzed for compounds that activated RORγ-dependent transactivation by more than 1.5-fold compared to the DMSO vehicle control, namely butyl-, hexyl-, heptyl- phenyl- and benzylparaben as well as benzophenone-6 and −10 (Fig. 2A and B). Concentration-dependent increases were observed for all compounds, with EC_50_ values for the three most potent RORγ agonists, *i.e.* hexylparaben, benzylparaben and benzophenone-10, of 144 ± 97 nM, 3.39 ± 1.74 μM and 1.67 ± 1.04 μM, respectively (Fig. 2B). The RORγ active compounds were subjected to XTT assays to assess cell viability and none of them showed any significant deviations from the vehicle control at the concentrations analyzed.

### 3.2. Competition of test compounds with the inverse agonist SR2211 and partial reversal of RORγ activity

RORγ typically shows constitutive activity due to the presence of endogenous agonists. This activity can be suppressed by inverse agonists, such as SR2211 (Kumar et al., 2012). To test whether the identified RORγ agonists can compete with the inverse agonist SR2211, basal RORγ activity was suppressed to approximately 35% by 1.25 μM SR2211. Cells were simultaneously exposed to SR2211 and a given test compound at different concentrations or vehicle control. All test compounds showed a concentration-dependent reversal of the SR2211-mediated inhibition of RORγ transactivation (Fig. 2C). A significant increase was obtained for hexylparaben, benzylparaben and benzophenone-10, consistent with our observations of the RORγ transactivation experiments in the absence of SR2211.

### 3.3. Structural similarity search to identify RORγ(t) modulators

**Fig. 3.**
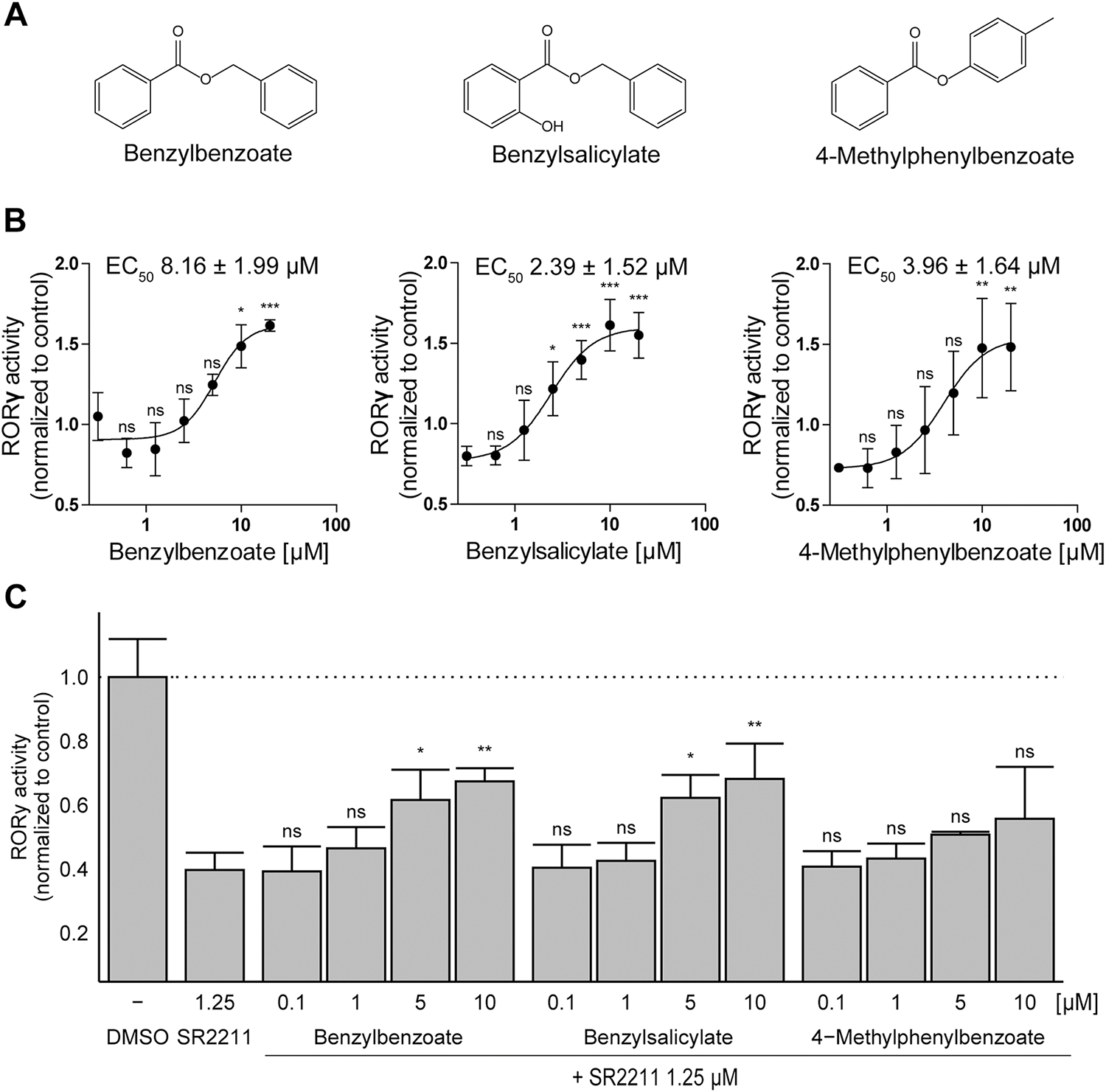
Analysis of three compounds selected from the structural similarity search for effects on RORγ activity. **(A)** Chemical structures of the three RORγ activators identified by structural similarity search. **(B)** Concentration-dependent activation of RORγ by the selected compounds. Concentration-response curves were fitted and analyzed by non-linear regression. **(C)** Activation of RORγ by the three selected compounds in the presence of a RORγ inverse agonist. RORγ expression was induced by doxycycline in the absence **(B)** or presence **(C)** of 1.25 μM SR2211. Cells were incubated with the test compounds at the concentrations indicated. Luciferase reporter activity was determined and normalized to that of the DMSO vehicle control. Data from three independent experiments represent mean ± SD. Data were analyzed by one-way ANOVA with Dunnett’s post-hoc test, p values: * < 0.05, ** < 0.01, and ns (not significant).

To identify additional chemicals present in cosmetic products that might interfere with RORγ(t) activity, a similarity search based on the structures of the newly identified parabens and benzophenone UV-filters and of the well-established RORγt agonist SR0978 (Chang et al., 2016) as templates was conducted against the CosIng cosmetics database. Already tested compounds (and their salts) were removed from the hit list, and the remaining chemicals were visually inspected and checked for their commercial availability, followed by *in vitro* testing of the most promising compounds to (Suppl. Table 1). The structures of all tested compounds and their activities towards RORγ are displayed in Supplementary Fig. 1. Benzylbenzoate, benzylsalicylate and 4-methylphenylbenzoate showed significant agonistic activity towards RORγ (Fig. 3A, B), with a concentration-dependent activation and EC_50_ values of 8.16 ± 1.99 μM, 2.39 ± 1.52 μM and 3.96 ± 1.64 μM, respectively (Fig. 3B). Cytotoxicity was excluded for all active compounds at the concentrations applied by performing XTT assays. Additionally, the ability of these three compounds to reverse the inhibition of RORγ activity by SR2211 was assessed (Fig. 3C). Whilst benzylbenzoate and benzylsalicylate were able to restore the suppressed RORγ activity in a concentration-dependent manner, 4-methylphenylbenzoate failed to do so and only tended to restore RORγ activity at the highest concentration of 10 μM.

### 3.4. Effect of hexylparaben, benzylparaben and benzophenone-10 on IL-17A and IL-22 mRNA expression in murine EL4 T-lymphocyte cells

**Fig. 4.**
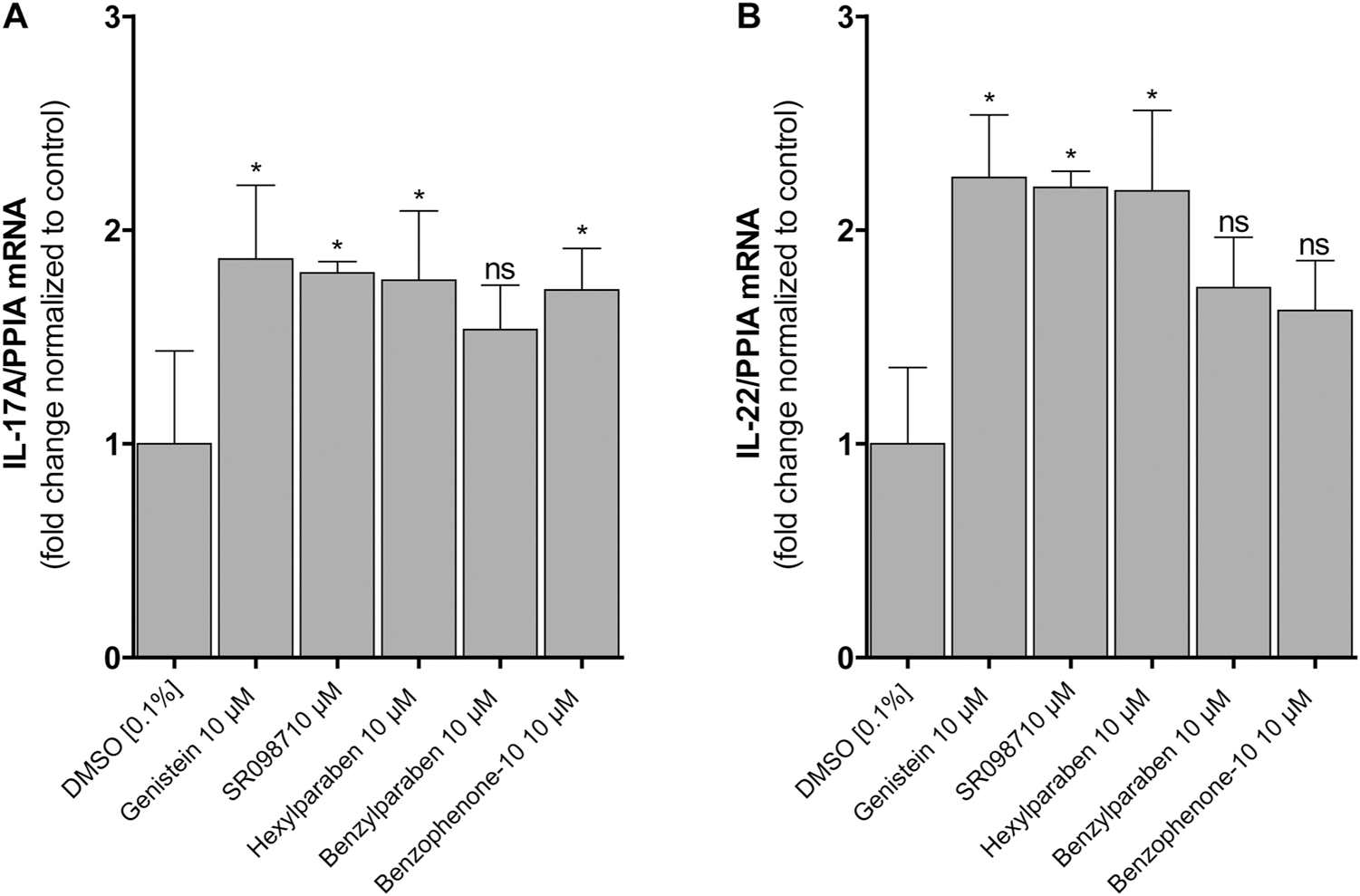
Effect of selected compounds on IL-17A and IL-22 mRNA levels in murine EL4 T-cells over expressing human RORγt. EL4 cells transfected with RORγt were stimulated with PMA/ionomycin to induce cytokine gene expression and exposed simultaneously to the compounds indicated. Subsequently, the mRNA expression of IL-17A **(A)** and IL-22 **(B)** were quantified by qRT-PCR and normalized to the expression of the housekeeping gene *PPIA*. Expression levels were normalized to vehicle control. Data represent mean ± SD from three independent experiments. Data was analyzed by one-way ANOVA with Dunnett’s post hoc test, p-value: * < 0.05, ns (not significant).

The murine EL4 T-lymphocyte cell line is commonly used to study effects on RORγt-dependent target gene expression and known to produce the pro-inflammatory interleukins IL-17A and IL-22. Based on their ability to activate RORγ and reverse the inhibitory effect of the inverse agonist SR2211 and their frequent use in consumer products, hexylparaben, benzylparaben and benzophenone-10 were analyzed for their effects on RORγt-dependent target gene expression in EL4 cells stimulated with PMA and ionomycin. It needs to be noted that the endogenous RORγt in the EL4 cells used in this study showed rather weak responsiveness and the increase of target gene transcription levels in the presence of known RORγt agonists was less pronounced than described in earlier studies (Chang et al., 2016; Kojima et al., 2015). Furthermore, the cells did not respond to specific RORγt inverse agonists; therefore, the human variant of RORγt was overexpressed in the EL4 cells to generate a more sensitive system. These cells allowed an efficient modulation of IL-17A and IL-22 transcription levels in EL4 cells by the known inverse agonist GSK2981278 (1 μM) and agonists SR0987 and genistein (10 μM). Exposure of these cells to hexylparaben, benzylparaben or benzophenone-10 resulted in an elevated expression of IL-17A and IL-22 (Fig. 4). qRT-PCR analysis revealed that all tested compounds, except benzylparaben for which a trend was detected, significantly increased IL-17A transcription at a concentration of 10 μM (Fig. 4A). With respect to IL-22, a significantly increased transcription was observed for the known RORγt activators genistein and SR0987 and for hexylparaben, whereas benzylparaben and benzophenone-10 tended to increase the transcription of this RORγt target gene (Fig. 4B). Benzylbenzoate, benzylsalicylate and 4-methylphenylbenzoate that were identified in the structural similarity search did not significantly enhance IL-17A and IL-22 mRNA transcription levels.

### 3.5. Agonistic effects of mixtures of parabens and benzophenone UV-filters towards RORγ

**Fig. 5.**
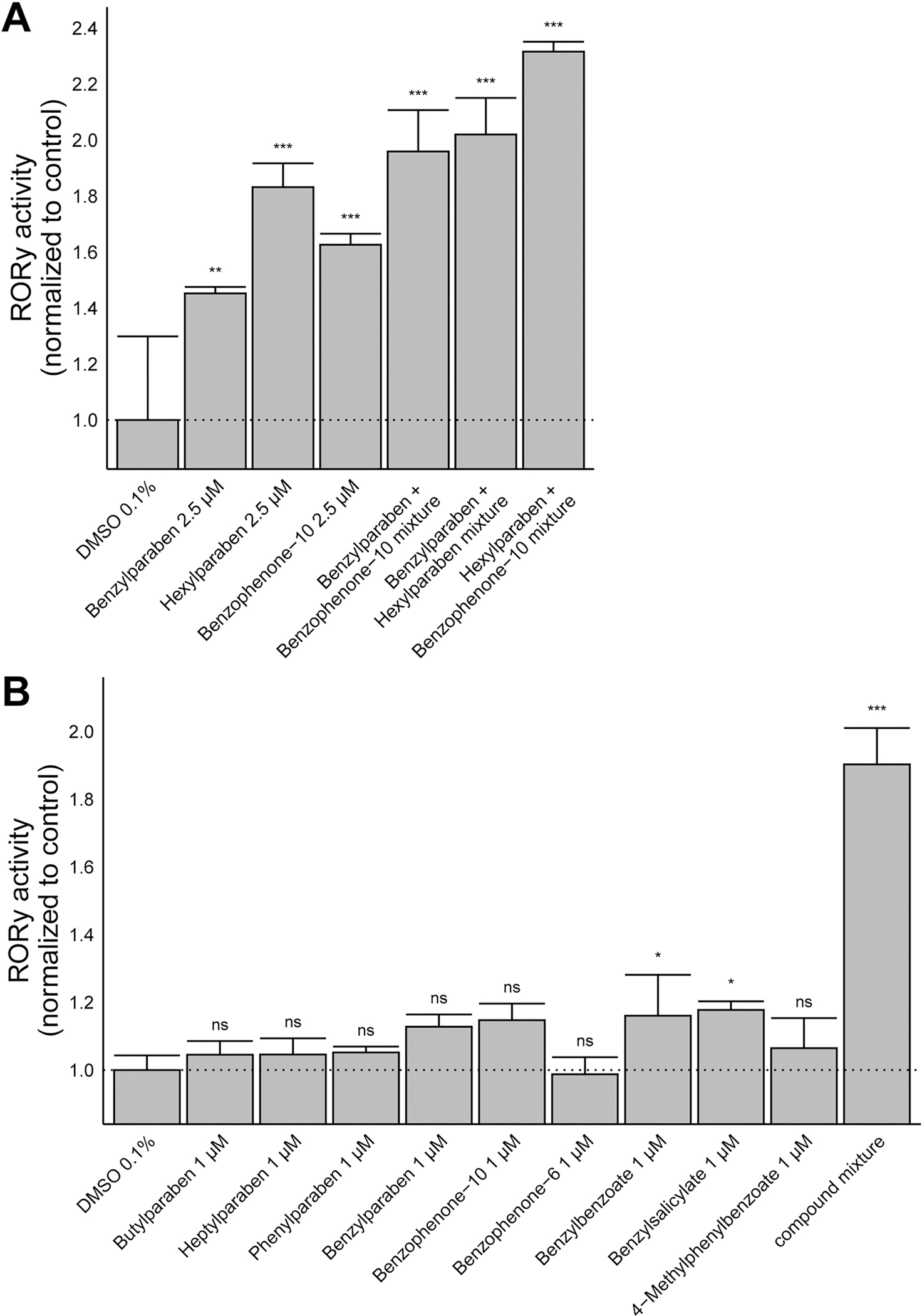
Mixture effects of parabens and benzophenone UV-filters on RORγ activity. RORγ expression was induced by doxycycline and cells were treated **(A)** with an individual test compound at a concentration of 2.5 μM or with mixtures of two compounds each at 2.5 μM, or **(B)** with an individual test compound at a concentration of 1 μM or a mixture containing all nine individual compounds each at 1 μM. Luciferase activity was measured and normalized to that of the DMSO vehicle control. Statistical analysis was performed by one-way ANOVA followed by the Dunnett’s post-hoc test. P values: * < 0.05, *** < 0.001, ns (not significant). Data represent mean ± SD from three independent experiments.

Parabens and UV-filters are usually added as mixtures of two or more compounds to cosmetics and sunscreens. To investigate whether mixtures of the identified parabens and benzophenone UV-filters exert more pronounced RORγ agonistic effects than the individual compounds, CHO RORγ Tet-on cells were exposed to either 2.5 μM of the most potent RORγ agonists benzylparaben, hexylparaben and benzophenone-10 alone or mixtures of two of them each at a concentration of 2.5 μM. As shown in Fig. 5A, all three mixtures led to a more pronounced activation of RORγ than the respective individual compounds. A comparison of the calculated sum of individual activities to the measured activity of the mixtures revealed no significant difference for two of the combinations (benzylparaben and benzophenone-10; benzophenone-10 and hexylparaben) while for one combination (benzylparaben and hexylparaben) the mixture showed somewhat lower effect than the calculated sum (Table 1). Additionally, to further explore mixture effects Tet-on cells were exposed to nine identified RORγ agonists alone at a concentration of 1 μM or to a mixture of all compounds each at 1 μM. It should be noted that the most active compound, hexylparaben, was excluded from this experiment as it may mask a possible additive effect of the other compounds due to its potent RORγ activation at concentrations well below 1 μM. As shown in Fig. 5B, the individual compounds at a concentration of 1 μM showed weak, *i.e.,* benzylbenzoate and benzylsalicylate, or no significant activation of RORγ. Importantly, treatment with the mixture containing each of these compounds at a concentration of 1 μM resulted in a strong activation of RORγ, comparable with that seen for the most potent compounds at a concentration of 10 μM (Fig. 2A, B and Fig. 3B). A comparison of the calculated sum of the nine individual agonistic effects to the effect of the mixture of the nine compounds revealed no significant difference, suggesting additive effects (Table 2).

## 4. Discussion

Parabens and UV-filters are major additives of cosmetics and body care products, enhancing product stability and shelf life, and these chemicals have raised considerable interest regarding their potential to disrupt endocrine functions (reviewed in Matwiejczuk et al., 2020; Wang et al., 2016). Whilst considerable research focused on the potential effects of such compounds towards estrogen and androgen receptors (Boberg et al., 2010; Darbre and Harvey, 2008; Kunz and Fent, 2006; Satoh et al., 2005; Schlecht et al., 2004), the present study, to our knowledge, is the first to investigate more closely a series of parabens (including their main metabolite *p*-hydroxybenzoic acid) and UV-filters for their effects on RORγ(t). Of the nine parabens and 15 UV-filters initially tested, the two parabens hexylparaben and benzylparaben and the UV-filter benzophenone-10 were identified as the most active RORγ agonists, with estimated EC_50_ values in the nano- and low micromolar range. Importantly, these substances were able to enhance the RORγt-dependent transcriptional expression of the pro-inflammatory cytokines IL-17A and IL-22, using the mouse T-lymphocyte cell model EL4.

IL-17A and IL-22 are produced by Th-17 cells and elevated levels of these cytokines are thought to be involved in the development and pathogenesis of autoimmune diseases, including psoriasis (Pan et al., 2013; Zheng et al., 2007). Due to the important role of RORγt in autoimmune diseases, this receptor is considered to be a promising drug target and several studies describe inverse agonists suppressing RORγt function, with the potential to treat patients suffering from such pathologies (Bronner et al., 2017; Fauber and Magnuson, 2014; Solt et al., 2010; Sun et al., 2019). In particular, autoimmune skin diseases are under investigation for the treatment with orally or dermally applied RORγt suppressors (Ecoeur et al., 2019; Imura et al., 2019; Sasaki et al., 2018; Smith et al., 2016; Takaishi et al., 2017). Th17 cells and IL-17 were also detected in skin biopsies of patients suffering from acne and have been associated with the development of this pathology (Agak et al., 2014; Kistowska et al., 2015). To our knowledge, there are currently no reports on effects of RORγt overstimulation by xenobiotics in such skin diseases. However, it can be expected to be unfavorable, because an upregulation of RORγt with elevated IL-17A and IL-22 levels has been proposed to play a role in immune disorders such as severe allergic asthma, psoriatic arthritis and UV-radiation induced promotion of skin cancer (Huang et al., 2020; Leijten et al., 2021; Lewis et al., 2021).

The consequences of human exposure to RORγt activating parabens and benzophenone UV-filters remain unclear and need to be carefully addressed. In 2014, following studies describing endocrine disrupting properties of parabens, the European Union restricted the maximal concentrations allowed in cosmetic products and prohibited the use of isopropyl-, isobutyl-, phenyl-, benzyl- and pentylparaben (EC, 2014). Therefore, human exposure to the newly identified RORγ agonist benzylparaben after the use of cosmetic products can be expected to be rather low. Hexylparaben, which has not been banned, has a relatively long alkyl side chain, resulting in reduced solubility. For this reason, this compound is less frequently used in cosmetics compared to the better soluble short-chain length parabens (CIR, 2008). Thus, due to the limited use, dermal exposure to hexylparaben (and the weakly active heptylparaben) can also be considered as low. Notably, butylparaben was detected in human plasma, seminal plasma, urine and breast cancer samples (Darbre et al., 2004; Frederiksen et al., 2011; Kang et al., 2016). However, the concentrations found in human samples were considerably lower than those found in the present *in vitro* study to activate RORγ. Nevertheless, the presence of unmetabolized parabens in human matrices demonstrated their uptake and exposure, despite the rapid hydrolysis to the RORγ inactive metabolite *p*-hydroxybenzoic acid. Parabens are metabolized by carboxylesterases that are highly expressed in the liver and intestine but also to a certain extent in the skin (Boberg et al., 2010; Jewell et al., 2007; Ozaki et al., 2013). Harville et al. (2007) showed by *in vitro* studies that the hydrolysis of parabens in human skin is much slower than in liver and the efficiency of hydrolysis was reduced with increased length of the alkyl chain. This suggests that longer chain parabens may accumulate in tissues such as subcutaneous fat. Whether extensive dermal application of cosmetics containing longer alkyl chain parabens might reach concentrations with the potential to disturb local RORγt activity remains unclear. The most widely used parabens in cosmetic products, methyl- and propylparaben (reviewed in Matwiejczuk et al., 2020) as well as ethylparaben, did not interfere with RORγ activity in the present *in vitro* study at much higher concentrations than what has been measured in human plasma samples (Calafat et al., 2010; Sandanger et al., 2011) suggesting that they may not disturb RORγt activity in the human skin.

Similar to parabens, maximal concentrations of UV-filters allowed in cosmetic products have been defined by the regulators as a consequence of the suspected endocrine disrupting effects of these substances (EC, 2009; FDA, 1999). Among the UV-filters tested in this study, benzophenone-10 showed the most potent RORγ agonistic effect. Allergic skin reactions have been reported after exposure to benzophenone-10 (Darvay et al., 2001; Goossens, 2016), furthermore a skin response involving Th17 cell activation (Peiser, 2013; Simon et al., 2014). Further investigations are required to test whether RORγt activation contributes to this adverse effect of benzophenone-10.

The RORγ active benzophenone UV-filters identified in this study are added typically in small amounts as stabilizing and protecting agents to cosmetics or plastic materials to improve the product’s shelf life (CIR, 2005). The expected exposure from contact with such products may well lie below the concentrations reported here to activate RORγ. However, it should be noted that for the RORγ agonistic compounds benzophenone-2 and −6 there are currently no studies in humans on plasma and/or urine levels available that would allow estimating bioavailability and potential activation of RORγ within skin after the use of cosmetics.

Amongst the benzophenone UV-filters showing agonistic RORγ effects, avobenzone and benzophenone-3 are allowed to be used as active UV-absorbing ingredients in sunscreens at high concentrations (up to 5% and 6%, respectively) (EC, 2009; FDA, 2019). Compared with avobenzone, which shows limited skin penetration and thus low bioavailability (Simeoni et al., 2004; Weigmann et al., 2001), benzophenone-3 has been reported to penetrate the human skin efficiently, and concentrations reaching the lower micromolar range in serum, urine and breast milk (304 μg/l, 300 μg/l, 779.9 ng/g milk) have been reported (Benson et al., 2005; Fernandez et al., 2002). Benzophenone-3 is metabolized in the human liver to benzophenone-1, benzophenone-8, 2,3,4-trihydroxybenzophenone, and 4,4’-dihydroxybenzophenone, which all were detected in human urine and breast milk samples (Janjua et al., 2008; Molins-Delgado et al., 2018; Tarazona et al., 2013; Watanabe et al., 2015; Yiin et al., 2015). While 4,4’-dihydroxybenzophenone was able to activate RORγ, benzophenone-1, showed only weak RORγ agonistic effect at micromolar concentrations. Whether other benzophenone-3 metabolites such as 2,3,4-trihydroxybenzophenone also can activate RORγt has not yet been assessed. Importantly, parabens and UV-filters are usually used as mixtures and as shown in this study, they can exert additive agonistic effects on RORγ(t). Whether benzophenone-3 and its metabolites may reach total concentrations within the skin and, less likely, within the body capable to activate RORγt and whether the low level RORγt overactivation by such xenobiotics impairs immune regulation and leads to adverse health effects requires further research.

4-(4-Methylphenylthio)benzophenone and 2,4,4’-trihydroxybenzophenone, included in the primary screening due to their structural similarity, are mainly used in printing inks (Bradley et al., 2013) and as UV stabilizers in plastic surface coatings of food packaging materials. 2,4,4’-trihydroxybenzophenone has been shown earlier to exhibit weak estrogenic activity (Suzuki et al., 2005). The present *in vitro* assessment found RORγ agonistic effects for both of these compounds at a concentration of 10 μM. However, the currently limited data on distribution and migration of these benzophenone UV-filters into foods as well as missing plasma compound levels do not allow a clear assessment regarding the possible modulation of RORγ activity in the intestine or in other organs, warranting further investigations.

The computational similarity search based on the newly identified RORγ activating scaffolds, followed by *in vitro* evaluation, revealed additional cosmetic additives with agonistic RORγ activity. Benzylsalicylate is added to cosmetic products as perfuming agent and light stabilizer (de Groot, 2020). Benzylbenzoate and 4-methylphenylbenzoate are fragrance ingredients and preservatives in cosmetic products (de Groot, 2020; Johnson et al., 2017). Estrogenic activity was reported earlier for benzylsalicylate and benzylbenzoate (Charles and Darbre, 2009); however, only at high micromolar concentrations that are unlikely reached in realistic human exposure scenarios. Both of these chemicals need to be declared when exceeding 0.001% in leave-on and 0.01% in rinse-off cosmetic products (de Groot, 2020). Interestingly, cosmetics-induced dermatitis was found to be associated with benzylsalicylate and benzylbenzoate; however, only in rare cases (Goossens, 2016). Among other cosmetic additives, benzylsalicylate is currently subjected to further inspections to evaluate its potential for endocrine disruption in consumers (EC, 2019). Notably, benzylbenzoate is also employed as an active ingredient in topical treatments for scabies (Haustein and Hlawa, 1989). As side effects of the therapy, skin irritation and allergic reactions were described. Further experimental investigations are needed to assess whether a chronic activation of RORγt might play a role in development of such side effects. It remains unclear whether dermal exposure to these chemicals can reach relevant systemic concentrations to activate RORγt and cause adverse effects. Furthermore, as described for benzylbenzoate (Johnson et al., 2017), these chemicals may also be hydrolyzed in the skin.

A structure-activity comparison of the analyzed parabens revealed that compounds with short side chains, such as methyl- and ethylparaben as well as the main metabolite *p*-hydroxybenzoic acid do not activate RORγ. Interestingly, RORγ-active parabens exhibit higher clogP values than inactive compounds (clogP values listed in Engeli et al., 2017), indicating that hydrophobic interactions may stabilize ligand binding. Analysis of the potential binding modes of the active hexylparaben and the inactive methylparaben in the RORγ orthosteric pocket by molecular docking predicted the phenyl-cores of both parabens to bind in the same location, while the prolonged alkyl chain of hexylparaben protruded into a sub-pocket containing several hydrophobic residues. These additional hydrophobic interactions are absent in case of methylparaben, providing an explanation for the distinct activity of these two compounds (Suppl. Fig. 2). In contrast to these two parabens, the binding analysis of the benzophenones studied was inconclusive and the interactions responsible for the differences between active and inactive compounds remain unclear. The prediction of the binding mode of these relatively small compounds within the ligand binding pocket of RORγ remains challenging and only permits initial insights into the ligand-protein interactions.

Even if the individual compounds identified may not reach concentrations *in vivo* to activate RORγ(t), it needs to be kept in mind that parabens and UV-filters are mainly used as mixtures in cosmetics and body care products. The agonistic mixture effects on RORγ identified in this study suggest additive effects of the newly identified compounds, which may be explained by their structural similarity and similar binding to the LBD of the receptor. However, because a cell-based assay was used, a contribution by synergistic action cannot be excluded. Follow-on studies using cell-free RORγ(t) assays should be performed to further corroborate the mechanism underlying the observed mixture effects.

The observed agonistic mixture effects on RORγ(t) emphasize the need for the development of extensive analytical methods to simultaneously quantify a larger number of relevant compounds in biological samples to assess exposure and test for correlations with biological functions. Additive and potentially also synergistic effects need to be taken into account in the safety considerations for the use of parabens and UV-filters. *In vivo* studies using animal models of autoimmune diseases may show whether the newly identified compounds and their metabolites alone or as mixtures can aggravate the pathophysiological effects by activating RORγt.

In conclusion, the present study revealed new scaffolds of parabens and benzophenone UV-filters as novel RORγ(t) agonists, with the ability to enhance pro-inflammatory cytokine expression in a mouse EL4 T-lymphocyte cell model. Furthermore, a fingerprint-based similarity search identified additional cosmetic compounds with agonistic effects towards this receptor. Further research needs to address the toxicological relevance of the identified activities towards RORγ(t), considering the rapid metabolism of parabens and the additive and potentially synergistic effects of parabens, benzophenone UV-filters and structurally related chemical additives in body care products.

## Supplementary Material

supplemental materials

## Figures and Tables

**Table 1:** Statistical comparison of the calculated sum and the measured effects of pairs of RORγ agonists of data shown in Fig. 5A.

Agonist [2.5 μM]	% increase in RORγ activity (mean ± SD)	Calculated sum of % increase in RORγ activity (mean ± SD)	Measured % increase in RORγ activity of the mixture (mean ± SD)

Benzylparaben	45 ± 2	108 ± 15	96 ± 5 (ns)
Benzophenone-10	63 ± 4		
Benzylparaben	45 ± 2	128 ± 8	102 ± 13 (*)
Hexylparaben	83 ± 8		
Benzophenone-10	83 ± 8	146 ± 11	132 ± 4 (ns)
Hexylparaben	63 ± 4		

RORγ activity was normalized to the vehicle control. The sums of RORγ activity of combinations of two agonistic compounds was calculated for each experimental replicate. Statistical analysis was performed by one-way ANOVA followed by the pairwise t-test with Bonferroni-adjusted *p* values. The calculated sums of RORγ activity of the two agonists were compared to the measured RORγ activity of their respective mixtures. *P* values:

* < 0.05

ns (not significant). Data represent mean ± SD from three independent experiments.

**Table 2:** Statistical comparison of the calculated sum and the measured effects of nine RORγ agonists of data shown in Fig. 5B.

Agonist [1 μM]	% increase in RORγ activity (mean ± SD)

Butylparaben	5 ± 4
Heptylparaben	5 ± 5
Phenylparaben	5 ± 2
Benzylparaben	13 ± 4
Benzophenone-10	15 ± 5
Benzophenone-6	−1 ± 5
Benzylbenzoate	16 ± 12
Benzylsalicylate	18 ± 3
4-Methylphenylbenzoate	6 ± 9
**Calculated sum of % increase in RORγ activity (mean ± SD)**	**81 ± 2**
**Measured % increase in RORγ activity of the mixture (mean ± SD)**	**90 ± 11 (ns)**

RORγ activity was normalized to the vehicle control. The sum of RORγ activity of all nine agonistic compounds was calculated for each experimental replicate. The calculated sum of RORγ activity of the nine agonists was compared to the measured RORγ activity of the respective mixture in a *t*-test. The two groups were not significantly different (ns). Data represent mean ± SD from three independent experiments.

## Abbreviations:

3-BC: 3-benzylidene camphor
4-MBC: 4-methylbenzylidene camphor
DMSO: dimethyl sulfoxide
ECFP: Extended Connectivity Fingerprint
IL-17A: interleukin-17A
IL-22: interleukin-22
ILC3: innate lymphoid cells 3
LBD: ligand binding domain
PCR: polymerase chain reaction
PMA: phorbol 12-myristate 13-acetate
PPIA: peptidylprolyl isomerase A
RORγ: retinoic acid-related orphan receptor gamma
Th17: T helper 17
