# Supplementary material for: Activation of retinoic acid-related orphan receptor γ(t) by parabens and benzophenone UV-filters
Source: Toxicology. Author manuscript; Available in PMC 2024 Apr 26. (PMC11046913; doi:10.1016/j.tox.2022.153159)
Supplement: supplemental materials [file NIHMS1981940-supplement-supplemental_materials.docx]

# Supplementary information

**Computational Methods**

To investigate binding modes of parabens and benzophenones within the orthosteric pocket of RORγ, the standard-precision docking protocol of Glide [1] retaining default settings were selected. The respective crystal structure (PDB ID: 6FZU) was retrieved from the Protein Data Bank [2] and processed using the Protein Preparation Wizard [3] within Maestro. There, hydrogen atoms were added, bond orders assigned, the protonation states of ionizable groups at pH 7.4 predicted, the hydrogen bonding network reoriented, and the system subjected to a restrained minimization with the OPLS3e force field at a convergence threshold of 0.3 Å for protein heavy atoms. The ligand conformers for docking were available from the previously conducted similarity search.

**Supplementary Table 1.** Compounds selected for in vitro testing including the 2D and 3D Tanimoto score compared to the template structure.

| **Template** | **Substance** | **CAS Nr.** | **Similarity-2D** | **Similarity-3D** |
| --- | --- | --- | --- | --- |
| Benzylparaben | Benzylnicotinate | 94-44-0 | 0.49 | 0.49 |
|  | Benzylbenzoate | 120-51-4 | 0.66 | 0.60 |
|  | Benzylsalicylate | 118-58-1 | 0.47 | 0.48 |
| Hexylparaben | Hexylbenzoate | 6789-88-4 | 0.64 | 0.62 |
| Heptylparaben |  |  | 0.60 | 0.49 |
| Butylparaben | Butylbenzoate | 136-60-7 | 0.61 | 0.59 |
| Phenylparaben | Phenylbenzoate | 93-99-2 | 0.63 | 0.56 |
|  | Phenylsalicylate | 118-55-8 | 0.43 | 0.43 |
|  | 4-Methylphenylbenzoate | 614-34-6 | 0.44 | 0.29 |
| SR0987 | Salophen | 118-57-0 | 0.19 | 0.21 |
|  | Acetaminophen | 103-90-2 | 0.17 | 0.20 |


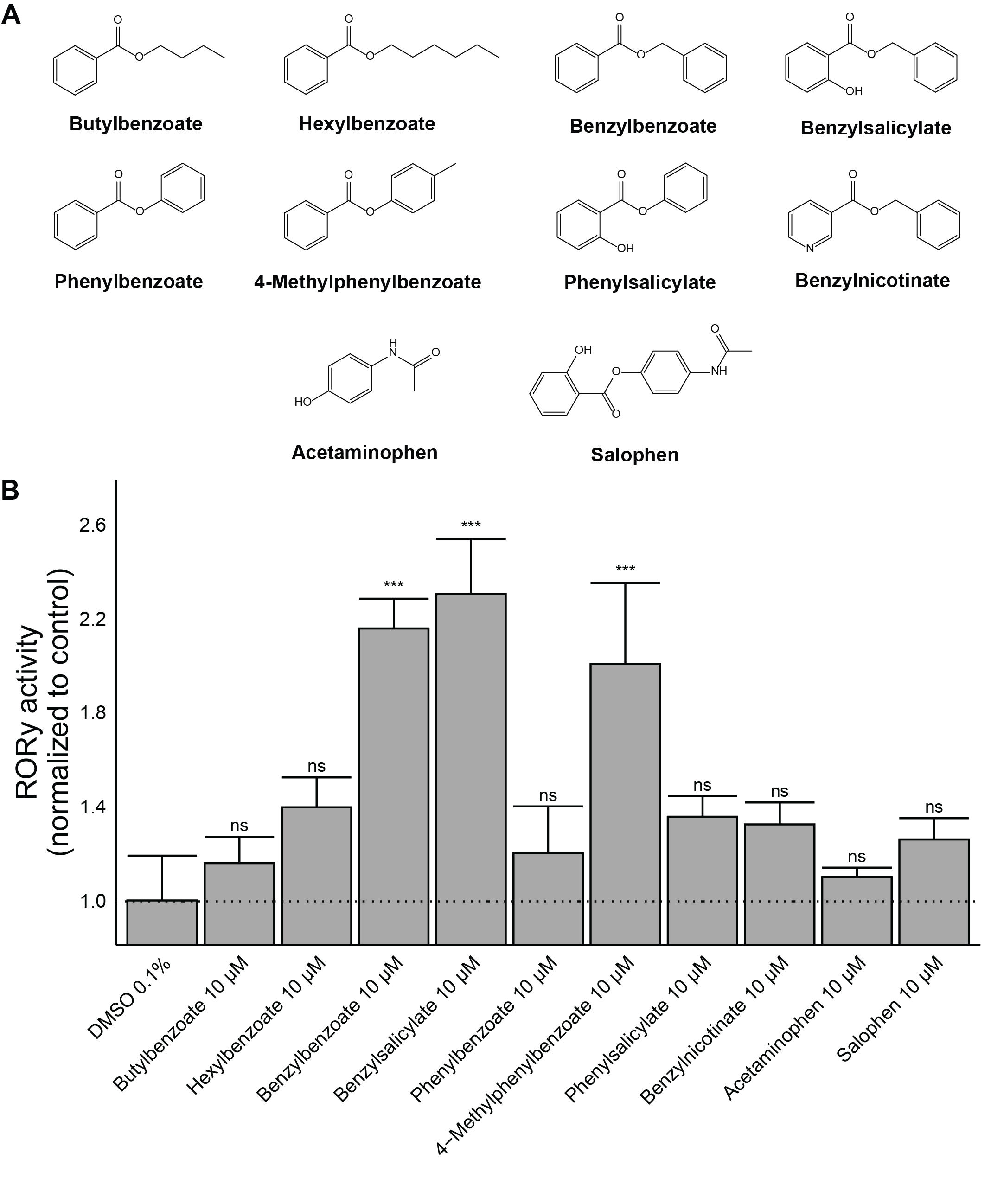


**Suppl. Fig. 1.** **Compounds selected from the similarity search. (A)** Chemical structures of investigated compounds from the similarity search. **(B)** RORγ activation by compounds selected from the similarity search. RORγ expression was induced by exposure of the Tet-on cells to doxycycline, and cells were incubated with 10 µM of test compounds. Luciferase activity was determined and normalized to that of the vehicle control DMSO. Data represent mean ± SD from three independent experiments. Data were analyzed by one-way ANOVA followed by the Dunnett’s post-hoc test, p values: * < 0.05, ** < 0.001, *** < 0.001, ns (not significant).


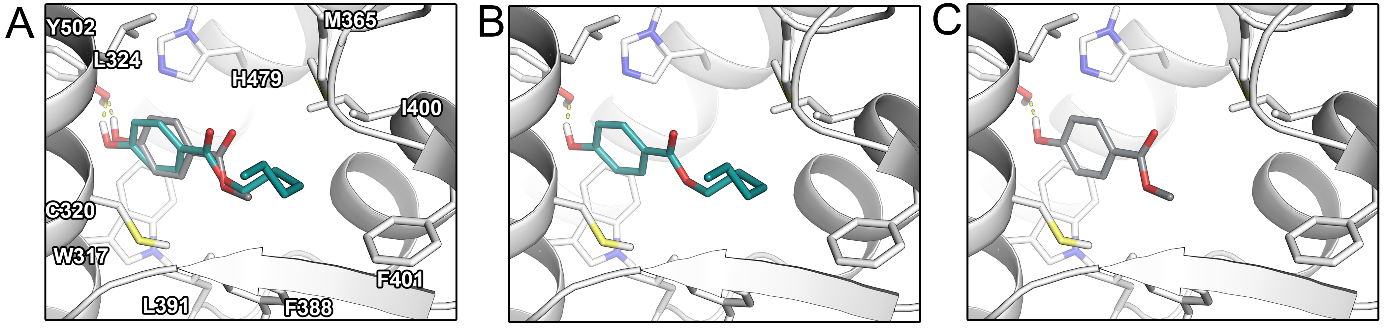


**Suppl. Fig. 2**. Predicted binding modes of **A)** methylparaben and hexylparaben together, **B)** hexylparaben, and **C)** methylparaben in the binding pocket of RORγ. Residues in the proximity of the ligand and hydrogen bonds to Y502 are depicted.

**Supplementary references**

[1] Halgren TA, Murphy RB, Friesner RA, Beard HS, Frye LL, Pollard WT, et al. Glide: A New Approach for Rapid, Accurate Docking and Scoring. 2. Enrichment Factors in Database Screening. J Med Chem. 2004;47(7):1750–9.

[2] H.M. Berman, J. Westbrook, Z. Feng, G. Gilliland, T.N. Bhat, H. Weissig, I.N. Shindyalov, P.E. Bourne. (2000) The Protein Data Bank Nucleic Acids Research, 28: 235-242.

[3] Madhavi Sastry G, Adzhigirey M, Day T, Annabhimoju R, Sherman W. Protein and ligand preparation: Parameters, protocols, and influence on virtual screening enrichments. J Comput Aided Mol Des. 2013;27(3):221–34.
